# Identifying typical trajectories in longitudinal data: modelling strategies and interpretations

**DOI:** 10.1007/s10654-020-00615-6

**Published:** 2020-03-05

**Authors:** Moritz Herle, Nadia Micali, Mohamed Abdulkadir, Ruth Loos, Rachel Bryant-Waugh, Christopher Hübel, Cynthia M. Bulik, Bianca L. De Stavola

**Affiliations:** 1 Department of Biostatistics & Health Informatics, Institute of Psychiatry, Psychology and Neuroscience, King’s College London, London, UK; 2 Population, Policy and Practice Research and Teaching Department, UCL Great Ormond Street Institute of Child Health, University College London, 30 Guilford Street, London, WC1N 1EH UK; 3 Department of Psychiatry, Faculty of Medicine, University of Geneva, Geneva, Switzerland; 4 Child and Adolescent Psychiatry Division, Department of Child and Adolescent Health, Geneva University Hospital, Geneva, Switzerland; 5 The Charles Bronfman Institute for Personalized Medicine, The Mindich Child Health and Development Institute, Icahn Mount Sinai School of Medicine, New York, NY USA; 6 Social, Genetic and Developmental Psychiatry Centre, Institute of Psychiatry, Psychology and Neuroscience, King’s College London, London, UK; 7 UK National Institute for Health Research (NIHR) Biomedical Research Centre, South London and Maudsley Hospital, London, UK; 8 Department of Medical Epidemiology and Biostatistics, Karolinska Institutet, Stockholm, Sweden; 9 Department of Psychiatry, University of North Carolina at Chapel Hill, Chapel Hill, NC USA; 10 Department of Nutrition, University of North Carolina at Chapel Hill, Chapel Hill, NC USA

**Keywords:** Growth mixture models, Latent class growth analysis, Longitudinal latent class analysis, Mixed effects models, ALSPAC

## Abstract

Individual-level longitudinal data on biological, behavioural, and social dimensions are becoming increasingly available. Typically, these data are analysed using mixed effects models, with the result summarised in terms of an average trajectory plus measures of the individual variations around this average. However, public health investigations would benefit from finer modelling of these individual variations which identify not just one average trajectory, but several typical trajectories. If evidence of heterogeneity in the development of these variables is found, the role played by temporally preceding (explanatory) variables as well as the potential impact of differential trajectories may have on later outcomes is often of interest. A wide choice of methods for uncovering typical trajectories and relating them to precursors and later outcomes exists. However, despite their increasing use, no practical overview of these methods targeted at epidemiological applications exists. Hence we provide: (a) a review of the three most commonly used methods for the identification of latent trajectories (growth mixture models, latent class growth analysis, and longitudinal latent class analysis); and (b) recommendations for the identification and interpretation of these trajectories and of their relationship with other variables. For illustration, we use longitudinal data on childhood body mass index and parental reports of fussy eating, collected in the Avon Longitudinal Study of Parents and Children.

**Electronic supplementary material:**

The online version of this article (10.1007/s10654-020-00615-6) contains supplementary material, which is available to authorized users.

## Introduction

Repeated observations of the same variable over time are increasingly frequent not only in purposely designed observational studies but also in large linked administrative health databases. In most applications, this type of data is analysed using mixed effects models [1, 2], leading to estimates of a population average trajectory, parametrised in terms of fixed effects, and the variation of the individual trajectories around this average. The latter is captured by the variances and covariances of subject-specific random effects. More recently, the focus of modelling such data has moved towards investigating whether there are multiple typical trajectories (see for example adolescent smoking [3], treatment response [4] and comorbidity [5]), leading to the characterisation of latent subgroups of individuals who share a common profile over time. Such groups are often referred to as “phenotypes” (e.g., early onset versus late onset of illness). Aiming to classify individuals into subgroups based on their longitudinal data has been described as being a person-centred approach, as opposed to the variable-centred approach typical of many regression analyses [6]. Often however these latent classes are studied in relation to explanatory variables [7–9] and/or later outcomes [10–12], and thus a person-centred classification may itself become a variable in a regression model, thereby blurring this distinction.

There are several modelling approaches that focus on identifying these trajectories, with alternative strategies available to relate them to earlier variables or later outcomes. The common feature of these approaches is that they all assume that a latent variable, composed of several classes, underlies the heterogeneity in how the variables evolve over time. These common approaches are:

1. Growth mixture models
2. Latent class growth analysis, also known as group-based trajectory models
3. Longitudinal latent class analysis

In this paper, we provide an overview of these three approaches and compare them in terms of assumptions, feasibility, and interpretation of the derived classes using mixed effects models as a reference. Another class of methods for the identification of latent trajectories are generalizations of cluster analysis (e.g., extentions of k-means clustering to longitudinal data [13]). As these methods do not invoke models, but rather rely on algorithms to classify individuals, they are not considered here. Their performance, however, has been found to be closely related to that of latent class growth analysis when trajectories vary smoothly with time [14].

To discuss the practical implications of adopting each of these modelling approaches above, and to illustrate how differences in resulting classes may derive, we analyse data derived from the Avon Longitudinal Study of Parents and Children (ALSPAC [15, 16]).

## Latent class trajectory models

### Mixed effects models

**Fig. 1:**
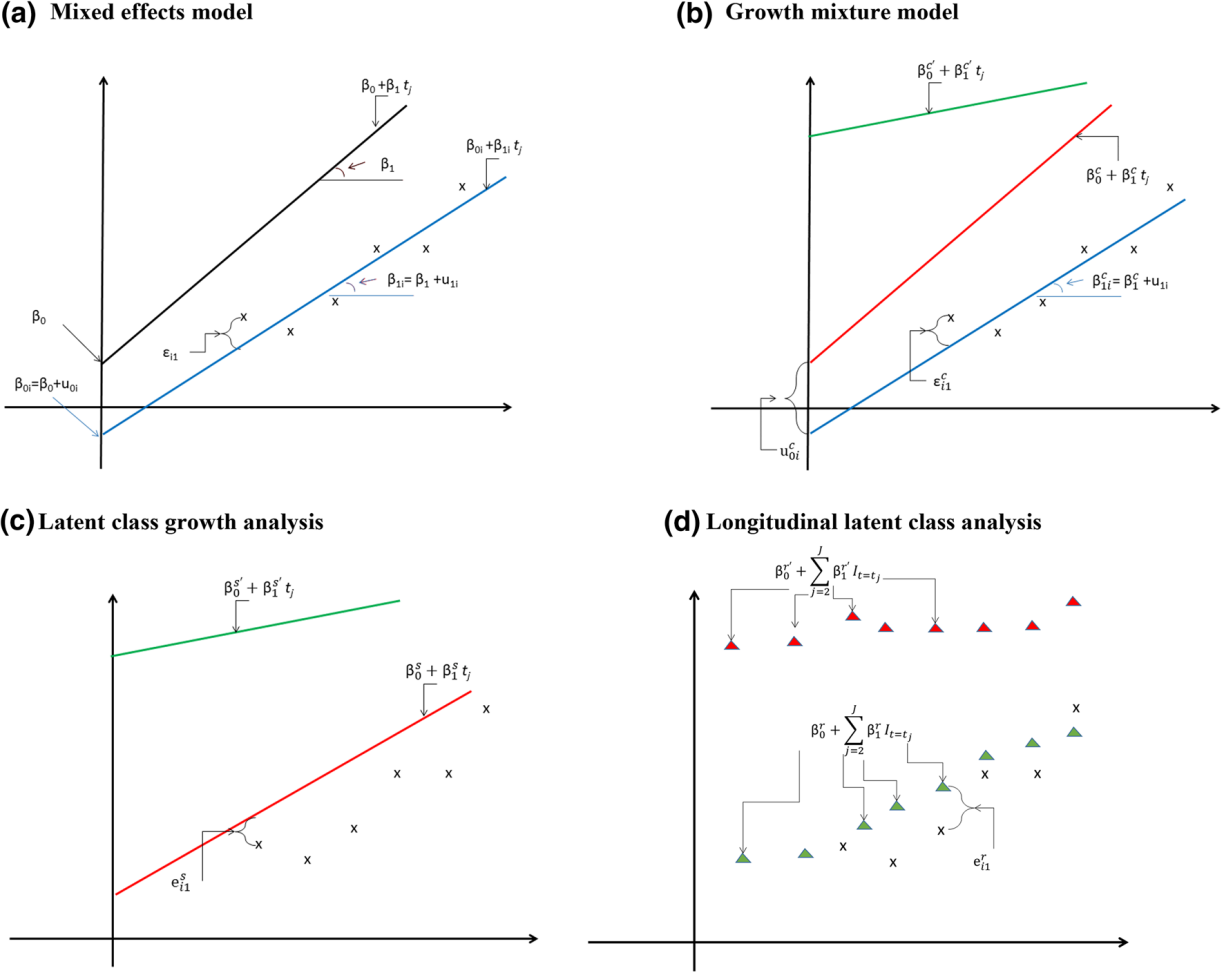
Graphical representation of alternative longitudinal models: **a** mixed effects model; **b** growth mixture model (GMM); **c** latent class growth analysis (LCGA); **d** longitudinal latent class analysis (LLCA). Black line: population mean trajectory; blue line: individual-specific trajectory; red and green lines: class-specific trajectories; red and green triangles: class-specific values; x: observations for individual *i*

Mixed effects models when applied to longitudinal data, relate outcomes collected on the same individual to their observation times, allowing for the shape of this relationship to vary across individuals. Consider a single outcome variable, $$ Z_{ij} $$, observed on individual *i* at times $$ t_{ij} $$, where *i* = 1, 2, …, *N*, and *j *= 0, 1, …, *J*. A typical specification of a mixed effects models for continuous outcomes, assuming a linear relationship with time, and the same observation times for all individuals, $$ t_{j} , $$ is

1 $$ Z_{ij} = {\beta_{0i}} + {\beta_{1i}} t_{j} + {\varepsilon_{ij}}, $$

where $\beta_{0i}$ and $\beta_{1i}$ are individual-specific coefficients, which have fixed ($\beta_{0}$ and $\beta_{1}$) and random ($u_{0i}$ and $u_{1i}$) components, with $\beta_{0i} = \beta_{0} + u_{0i}$ and $\beta_{1i} = \beta_{1} + u_{1i}$. The fixed coefficients $\beta_{0}$ and $\beta_{1}$ are shared by all individuals, while the error terms $\varvec{u}_{i} = \left( {u_{0i} ,u_{1i} } \right)$ are unobserved random variables that capture the individual departures from the population average trajectory, $\left( {\beta_{0} + \beta_{1} t_{j} } \right)$. The error terms $\varvec{u}_{i}$ are usually assumed to be jointly normally distributed with mean zero and free covariance matrix $\varvec{ }\Omega _{\bf u} ,$ and the residual errors $\varepsilon_{ij}$ to be independently and normally distributed, conditionally on $\varvec{u}_{i}$ and t, with constant variance $\sigma_{\varepsilon }^{2}$. The $\varepsilon_{ij}$ capture the distance between the observed data for the *i*-th individual to the true individual-specific trajectory, $(\beta_{0i} + \beta_{1i} t_{j} )$ (Fig. 1a). Here we consider $t_{j}$ to indicate the actual observation time, so that the relationship with time is properly captured. When information is gathered in terms of waves, as in panel data, we would recommend translating this information into an appropriate time-scale.

When $Z_{ij}$ is an ordered categorical variable, with (K + 1) categories, a mixed effects model is usually specified in terms of a latent continuous variable $Z_{ij}^{'}$ specified as

2 $$Z_{{ij}}^{'}  = \beta _{{0i}}  + \beta _{{1i}} t_{j}  + \epsilon_{{ij}} , $$

where $\beta_{0i}$ and $\beta_{1i}$ are defined as before but with the independent error $\epsilon_{ij}$ following a logistic distribution with mean 0 and variance $\frac{{\pi^{2} }}{3}$ (where $\pi \; {\text{is}}\; {\text{the}}$ constant representing the ratio of a circle’s circumference over its diameter). The observed categorical variable $Z_{ij}$ is assumed to have been generated from this latent variable according to unobserved cut-points (“thresholds”) $\tau_{k}$, *k *= *1, …, K,* with $Z_{ij} = 1$ if $Z_{ij}^{'} \le \tau_{1}$; $Z_{ij} = 2$ if $\tau_{1} < Z_{ij}^{'} \le \tau_{2}$; …; $Z_{ij} = \left( {K + 1} \right)$ if $Z_{ij}^{'} > \tau_{K}$. The thresholds are the expected values of the latent variable $Z_{ij}^{'}$ at which an individual transitions from a value *k* to a value (*k *+ 1) on the categorical outcome variable $Z_{ij}$.

Generalisations of models (1) and (2) that include non-linear relationships with time are straightforward, likewise models where the coefficients for these additional non-linear terms include random components, as in

3 $$ Z_{ij} = \beta_{0i} + \beta_{1i} t_{j} + \beta_{2i}  {t_{j}^{2}} + \varepsilon_{ij} . $$

Estimation is generally by maximum likelihood (ML, or restricted maximum likelihood when the study is small [17]), with the estimation-maximisation algorithm used in the presence of missing outcome data under the missing at random (MAR) assumption [18].

**Fig. 2:**
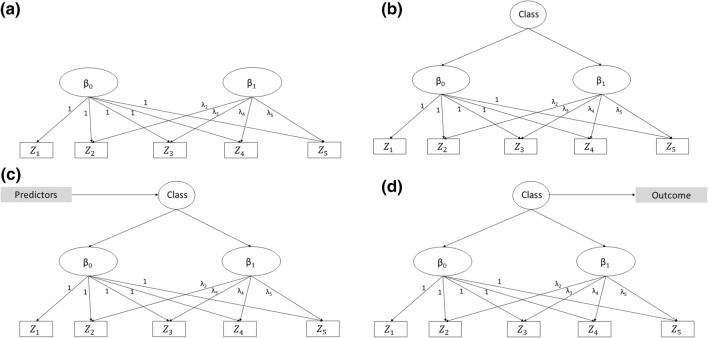
Structural equation modelling representation of: **a** mixed effects model; **b** growth mixture model; **c** growth mixture model with predictors; **d** growth mixture model with distal outcome

When individuals are observed at the same times $t_{j}$, as assumed here, there is an alternative formalization of mixed effects models that arises from to the confirmatory factor analysis framework (and, more generally, the structural equation modelling [SEM] literature). This framework views the random coefficients of a mixed effects model as latent factors, “manifested” by the joint distribution of the longitudinal observations, $\varvec{Z}_{i} = \left( {Z_{i1} , Z_{i2} ,, \ldots ,Z_{iJ} } \right)$ [19]. Model (1) for example could also be written as

4 $$ Z_{ij} = \beta_{0i} + \lambda_{j}  \beta_{1i} + \varepsilon_{ij} , $$

where $\beta_{0i}$ and $\beta_{1i}$ are the original individual-specific coefficients that are now viewed as latent variables. The regression coefficients $\lambda_{j}$ (referred to as “factor loadings” in the SEM literature) are not estimated but are pre-determined according to the timing of the observations. For model (1) the factor loadings would be: $\lambda_{1} = 0,  \lambda_{2} = \left( {t_{2} - t_{1} } \right),  \lambda_{3} = \left( {t_{3} - t_{2} } \right),$ etc. This representation of model (1) can be viewed graphically in Fig. 2a, where the factor loadings are shown above the arrows linking the latent individual-specific coefficients to the observed data. Adopting this approach has several advantages, in particular the option of using SEM software for estimation, and also extending the model for example by allowing the error terms $\varepsilon_{ij}$ to have time-specific variances, $\sigma_{\varepsilon j}^{2}$, or more complex extensions as discussed below.

### Growth mixture models

Growth mixture models assume that there are multiple mixed effects models, each representing a subgroup (i.e. “class”) of trajectories that share a common mean and shape (with, potentially, class-specific error variance structures) [20, 21]. Growth mixture models are therefore generalisations of mixed effects models (Fig. 1b).

Formally, they are specified as follows. Let *C* indicate the number of latent classes in the population, distributed with probabilities $p_{c}$, *c *= 1,…*, C,* with $0 \le p_{c} \le 1$ and $\mathop \sum \nolimits_{c = 1}^{C} p_{c} = 1$ [22]. As the latent classes are unknown, we model the observed data using as a mixed effects model specific to the latent class *c* each individual belongs to, with the joint distribution of the data then being a mixture of these distributions, weighted by the probability of each class, $p_{c}$. For example, a growth mixture model generalisation of model (1) is,

5 $$ Z_{ij|c} = \beta_{0i}^{c} + \beta_{1i}^{c}  t_{j} +  \varepsilon_{ij}^{c} , \quad  {\text{for}}\;c = 1, \ldots ,C, $$

where ${\beta_{0i}^{c}} = {\beta_{0}^{c}} + {u_{0i}^{c}}$, ${\beta_{1i}^{c}} = {\beta_{1}^{c}} + {u_{1i}^{c}}$, $\varvec{u}_{i}^{c} = \left( {  {u_{0i}^{c}}, {u_{1i}^{c}}} \right)$ and ${\varepsilon_{ij}^{c}}$ are defined as before, although specifically for each class *c*. The graphical representation of this model is shown in Fig. 2b. Assuming that all classes have the same error structure may be unrealistic; therefore class-specific covariances $\Omega_{\varvec{u}}^{\varvec{c}}$ for the individual-level error terms are often considered.

For categorical variables, we would specify $Z_{ij|c}^{\prime} = {\beta_{0i}^{c}} + {\beta_{1i}^{c}}  {t_{j}} + {\epsilon_{ij}^{c}}$, with ${\epsilon_{ij}^{c}}$ following a logistic distribution.

Because the number of classes is unknown, the estimation is carried out conditionally on a pre-specified number of classes. Estimation is by Maximum likelihood (ML) with the expectation–maximization (EM) algorithm because the classes are unobserved [23]. As several local maxima for the likelihood are expected to be found with such complex models, multiple starting points for the estimation routine are recommended, before maximization is deemed to have been reached [19]. Following estimation, posterior class probabilities can be derived and used to assign individuals to classes according to their largest value (“modal assignment”), or to weigh individuals when calculating predicted class frequencies.

In order to identify the number of classes that best fits the data, a number of goodness-of-fit criteria are compared. Those commonly recommended in the literature [24] are the Akaike Information Criterion (AIC), the Bayesian Information Criterion (BIC), and its sample size-corrected version (c-BIC). For each of these, lower scores indicate (relatively) better fitting models. The parametric bootstrap likelihood ratio test (BLRT) has also been recommended as an additional comparative tool given its performance in simulations [25]. However it is disadvantaged by being computationally intensive and affected by poor performance in small samples [19]. These goodness-of-fit criteria do not necessarily agree, in the sense that they may not all point to selecting the same model. Hence, additional considerations are often invoked, such as interpretability of the latent trajectories, and the avoidance of too small classes (e.g. < 5% of the study population) that may lead to lack of reproducibility of the results.

The quality of the classification of a model, the so-called “entropy”, is also often reported, with values close to 1 indicating good classification. Specifically, this is a summary measure that captures how well class membership is predicted given the observed outcomes. However, this interpretation requires the model to be correct, and thus entropy values should not be overinterpreted [25].

As described, these criteria are applied sequentially on models with increasing numbers of classes using the same dataset. It has been suggested that cross-validation should be used instead [26]. This would involve fitting the model with a given number of classes on a subset of the data, followed by using the selected model on the remaining data and assessing its goodness of fit. A more sophisticated version of this would involve k-fold cross-validation. This approach, however, requires larger datasets than those usually available in typical epidemiological studies and would still depend on which goodness of fit criterion is used.

### Latent class growth analysis

Latent class growth analysis [27] specifies models that are similar to growth mixture models. However, latent class growth analysis models assume *no* individual-level random variation within each class, and therefore individuals assigned to the same class share exactly the same trajectory.

Formally, latent class growth analysis specifies models with the same structure as model (5) but with fixed effects regression coefficients, albeit specific to each class. Denoting a latent class growth analysis class by *s*, this model is expressed as,

6 $$ Z_{ij|s} = \beta_{0}^{s} + \beta_{1}^{s}  t_{j} +  e_{ij}^{s} ,\quad s = 1, \ldots ,S $$

where *Z* is a continuous variable and $e_{ij}^{s}$ are independently distributed error terms. Because there is no within-cluster variation (i.e. there are no $\varvec{u}_{i}^{s}$ and the class-specific coefficients $\beta_{0}^{s}$ and $\beta_{1}^{s}$ are the same for every member of class *s*), these error terms capture random perturbations of each observed data point from their class specific trajectory (Fig. 1c). The assumption that these errors are independently distributed, as implicit in most software [28, 29], may be unrealistic however as one would expect individual trajectories that belong to the same class to be heterogeneous and the individual-specific departures from the class-specific trajectories to be correlated. Departures from this assumption can have consequences, as discussed in “Assumptions”.

### Longitudinal latent class analysis

These models are a variation of latent class growth analysis models that ignores the longitudinal nature of the data. The model for an individual belonging to the longitudinal latent class *r* is specified as,

7 $$ Z_{ij|r} = \beta_{0}^{r} +  \mathop \sum \limits_{j = 2}^{J}   \beta_{j}^{r}  I_{{t = t_{j} }}^{{}} +_{{}}  e_{ij}^{r} ,\quad r = 1, \ldots ,R, $$

where $I_{{t = t_{j} }}^{{}}$ are dummy (0/1) indicators of the times when $Z_{ij}$ is observed (Fig. 1d). Hence, latent classes are identified without exploiting the information on the time order of the observations, but also without forcing any parametric relationship between the outcomes and time.

### Comments

#### Assumptions

Mixed effects models, growth mixture models, and latent class growth analysis rely on parametric assumptions for the relationship between the observed outcomes and time. These models, together with longitudinal latent class analysis, rely on distributional assumptions for the error terms. Mixed effects models and growth mixture models make additional assumptions regarding the within-subject correlations (parametrized by $\varvec{ }\Omega _{\bf u}$ and $\Omega _{\varvec{u}}^{\varvec{c}}$, respectively). Violations of these assumptions have different consequences depending on the type of outcome and modelling approach. Misspecified distributions and correlation structures in mixed effects models do not impact on the consistency of the fixed effect estimates when the observed outcomes are continuous, but they may bias inferences [1]. If the outcomes are categorical, however, bias will affect the fixed effects estimates as well [1, 30]. Non-parametric specifications of the random effect distributions have been proposed to deal with these issues [31], as described below.

The impact of these misspecifications may also influence the estimated number of classes of a growth mixture model. If, the assumed covariance structure is too simple, the number of classes may be greater because more are needed to capture the variability in the data [32]. For this reason, and as demonstrated in simulations [33], when selecting the number of classes for growth mixture models, one should in principle allow for general specifications, e.g., with class-specific covariance matrices $\Omega _{u}^{c} ._{{}}$ and time-specific residual error variance $\sigma_{\varepsilon j}^{2}$ [33]. How general these matrices can be, will be limited by the study size and may not be suitable with binary outcome data when their prevalence is low [33].

The assumption of independence for the residual errors $e_{ij}^{s}$, conditional on class *s*, which is usually made when performing latent class growth analysis, is most likely to be incorrect, especially when there are several observations per individual. Violations may lead to biased estimates of the class-specific regression coefficients [33] unless the classes are well separated, e.g. entropy > 0.8 [32]. Such bias is more prominent when the true covariance structure is complex, the study size is small (< 500), or the outcomes are binary [30].

Another assumption often made with longitudinal data is that of the outcome data being missing at random (MAR) [18]. This assumes that the propensity of missing an observation, possibly because of an individual dropping-out of the study, depends on the observed data only. If met, model estimation by ML (for mixed effects models), or ML with EM (for growth mixture models), based on incomplete data is not affected by selection bias [17, 34]. It is often the case, however, that missingness depends on other variables, most commonly social factors. In such circumstances, one could include the predictors of missingness in the model, as discussed in “Relating latent classes to earlier explanatory variables or to later outcomes”.

#### Interpretation

In interpreting the results of whichever approach, one has to take into consideration all of the issues described above. Of note is that latent class growth analysis models were initially proposed as a semi-parametric version of mixed effects models where the variation in trajectories around a single class is approximated by a number of fixed trajectories, as opposed to assuming jointly normally distributed random effects [35]. In other words, the classes are used to capture the overall variation so that, when the data are truly from a mixture of K classes (as in growth mixture models), a larger number of classes will be needed to extract the main features of the data when adopting latent class growth analysis [23, 27]. Thus, interpreting the resulting classes as if they had a theoretical underpinning would be inappropriate in most settings. In contrast, growth mixture models distinguish the typologies represented by the latent classes from the within-class variation. Again, however interpretation should be cautious because of their stronger parametric assumptions.

#### Analytical strategy

These observations highlight the need for a comprehensive set of model specifications to be considered and then compared, ranging from single class mixed effects models to growth mixture models and then latent class growth analysis and longitudinal latent class analysis models, before concluding whether there are multiple trajectory types and what they capture.

As a first step, we would recommend fitting the most general mixed effects model that the data can identify in order to investigate the extent of between-individual heterogeneities. The distributions and correlations of the predicted random effects from such a model could then be used to aid the interpretation of the best fitting growth mixture and best fitting latent class growth analysis (or longitudinal latent class analysis) models. Comparing the classes predicted from these different model specifications, numerically and/or graphically, would also help clarify whether similar typologies emerge when adopting different modelling approaches.

If, even after allowing for the fact that some of the classes from a latent class growth analysis model actually will aim to capture the distribution of individual trajectories within a particular “true” class, little agreement is found, one should investigate whether model misspecifications might explain the discrepancies. As discussed in “Assumptions”, these may lead to biased parameter estimates and/or incorrect selection of the number of classes. Examination of the distributions of the estimated time-specific residuals derived for each class might indicate for example that the model is not properly reflecting the data if they were found to be skewed. This would happen for example if the relationship with time were misspecified in one of the classes.

#### Relating latent classes to earlier explanatory variables or to later outcomes

Once classes are derived, it is possible to relate them to earlier explanatory variables or later outcomes. Any inferences drawn on these relationships, however, should account for the fact that the classes are not directly observed but derived under certain modelling assumptions. There are two main approaches to achieve this.

The first approach—the “1-step approach”—consists of extending the original model for the latent trajectories to include associations with the explanatory or the later outcome variables of interest. This is easily achieved within an SEM framework (Fig. 2c, d), with the joint estimation of the latent classes and their relationship with other variables (respectively the “measurement” and “structural” parts of the SEM model) accounting for the uncertainties of class assignment.

The second commonly used approach breaks down the estimation into three steps (“3-step approach”). The best fitting latent trajectory model is fitted (1st step) and then used to assign individuals to their most likely class using the predicted posterior probabilities of belonging to each class (2nd step). These classifications are then included as outcomes or predictors in the relevant new analyses, accounting for the uncertainty of the classification performed in step 2 (via the probabilities of the true class given the assigned class estimated in step 1) [36].

The first approach is not generally recommended when the aim is to relate explanatory variables to the latent classes because the identification of the latent classes is potentially affected by which variables are included in the model [37]. One exception to this concern is when the reason for including the covariates in a 1-step analysis is to meet the MAR assumption when the longitudinal outcome data are affected by missingness. In this case, one would want to condition on these covariates to avoid the bias that would arise from analysing incomplete data.

More serious concerns arise when relating latent classes to a later outcome, because in the latter case the outcome has the same direction of association with the classes as the longitudinal variables that lead to their identification (see Fig. 2d; [36]).

When the entropy of the latent class model is greater than 0.80, results from the 1- or 3-step approach have been found to be similar [36]. In practice, however, the 1-step approach may be unfeasible, especially when the longitudinal data are categorical, so that the 3-step approach should be adopted (with multiple imputation if missingness depends on covariates, and with the selection of the number of classes made from the most frequently best solution among the imputed sets).

## The ALSPAC study

### Participants

We analysed data from the Avon Longitudinal Study of Parents and Children (ALSPAC), a population based, longitudinal cohort of mothers and their children born in the southwest of England, to illustrate the different modelling strategies. Details of the study are given elsewhere [15, 16]. Briefly, all pregnant women expected to give birth between the 1st April 1991 and 31st December 1992 were invited to enrol in the study. From all pregnancies (n = 14,676), 14,451 mothers opted to take part, and 13,988 of their children were alive at 1 year. Analyses are restricted to girls only for simplicity, after randomly selecting one child per set when birth was from a multiple pregnancy. Please note that the study website contains details of all the data that are available through a fully searchable data dictionary and variable search tool: http://www.bristol.ac.uk/alspac/researchers/our-data/.

### Variables

#### Longitudinal variables

We aimed to model the repeated measures of a continuous and an ordinal variable:

- Body mass index (BMI; in kg/m^2^), objectively measured up to six times when participants were (around) 8, 10, 11, 12, 13, and 16 years. Height was measured to the nearest millimetre with the use of a Harpenden Stadiometer (Holtain Ltd.). Weight was measured with a Tanita Body Fat Analyzer (Tanita TBF UK Ltd.) to the nearest 50 g.
- Parental reporting of fussy eating consisted of responses to the question “How worried are you because your child is choosy?” for which there were three possible answers: “No/did not happen”, “Not worried”, and “A bit/greatly worried”. These were observed up to eight times during the first ten years of life, specifically at around 1.3, 2.0, 3.2, 4.6, 5.5, 6.9, 8.7, and 9.6 years. A more detailed description of these data can be found in Herle et al. [38].

#### Explanatory variable

Birth weight (in kg) was used as the explanatory variable of interest in our examples. This variable was available on 4462 (99%) girls among those with at least one longitudinal BMI and on 5750 (99%) girls with at least one fussy eating measurement. Mean birth weight was 3.37 kg (SD = 0.51) and 3.36 kg (SD = 0.51) in these two subgroups. It was internally standardized using these means and SDs in the analyses.

#### Later outcome

Body fat mass index (FMI) [39] at age 18 years was the later outcome of interest. It was defined as the ratio of total body fat mass (in kg) over height (in metres) squared. Body fat was objectively measured using the Tanita Body Fat Analyser (Model TBF 401A) and height as described above. Data on FMI were available on 2443 (55%) girls with at least one longitudinal BMI measurement and 2464 (42%) girls with at least one longitudinal fussy eating measurement. Mean FMI was 21.57 kg (SD = 9.56) and 21.62 kg (SD = 9.52), respectively.

#### Computer code

Examples of Mplus and Stata code used for these analyses can be found in https://github.com/MoritzHerle/Identifying-typical-trajectories-in-longitudinal-data. Some of these analyses can also be performed in R (with the lcmm package); the relevant code can also be found in this depository.

#### Ethics

The authors assert that all procedures contributing to this work comply with the ethical standards of the relevant national and institutional committees on human experimentation and with the Helsinki Declaration of 1975, as revised in 2008. Ethical approval for the study was obtained from the ALSPAC Ethics and Law Committee and the Local Research Ethics Committees.

### Data description

**Fig. 3:**
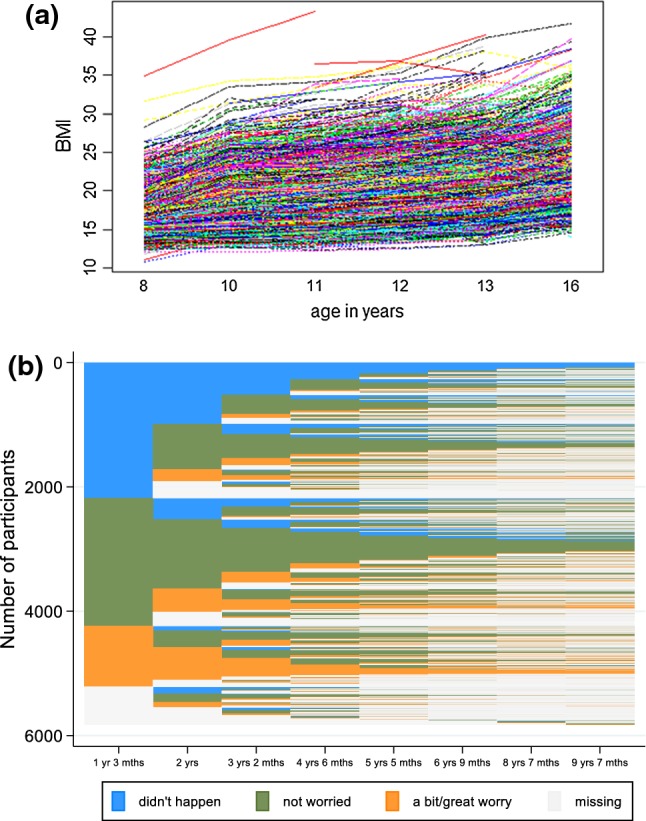
Observed trajectories in **a** body mass index (BMI; kg/m^2^), N = 4571 and **b** fussy eating, N = 5824, Avon Longitudinal Study of parents and children

Figure 3a shows the observed individual BMI trajectories for all participants with at least one BMI observation (“spaghetti plot”), while Fig. 3b shows the equivalent plot (“lasagne plot”) for the categorical fussy eating variable, with a change in colour along time representing a change in category. Both variables show considerable and increasing variation over time, as well as an increasing frequency of missing data. Details of the completeness of the longitudinal BMI data are given in Supplementary Tables 1 and 2; they highlight that the majority of the participants included in these analyses had six data points and are therefore quite complete. A total of 4517 girls had at least one longitudinal BMI measure and 5824 girls had at least one longitudinal parental report of fussy eating. In the following, we assume that MAR was satisfied and included in the analyses all girls with at least one longitudinal observation of the relevant outcome variable.

## Longitudinal phenotypes

### BMI

#### Mixed effects models

As a first step, we fitted mixed effects models to the longitudinal BMI measures, with age capturing the dependence on time. Given the observed trajectories, we specified models that included a linear and quadratic term for age and correlated random effects for the intercept and the two slopes of the linear and the quadratic age term, before considering simpler specifications. The resulting best fitting model had random intercepts, and random coefficients for the linear and the quadratic age term (with freely estimated covariances and residual variances; details in Supplementary Table 3).

#### Growth mixture models

**Fig. 4:**
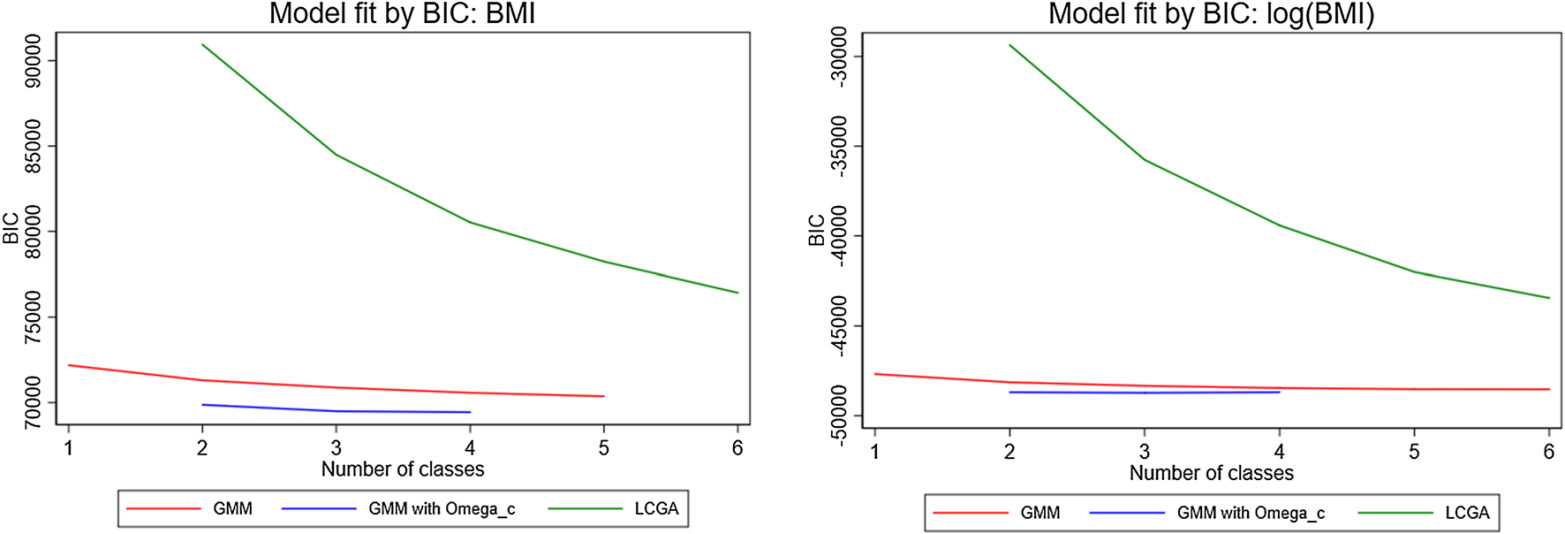
Bayesian information criterion (BIC) by number of classes for different specifications of the growth mixture model (GMM) (with/without homogeneous within-individual correlation matrix, Ω_c_) and of the latent class growth analysis (LCGA) model for body mass index (BMI) and log(BMI)

Mixed effects models can be viewed as single class growth mixture model. Hence, their goodness of fit can be compared with that obtained from growth mixture models with increasing numbers of classes, allowing for class-specific covariance structures [33]. We compared growth mixture models with up to six classes using the criteria described in “Growth mixture models”. Among the models that assumed the same error structure across classes, the three-class solution fitted the data best according to the AIC, BIC and c-BIC (red and blue line in Fig. 4, Supplementary Table 4). Relaxing this assumption led to improvements in these indices but led to substantially worse entropy (it dropped from 88.9% to 57.5% for the (best) 3-class solution) and to some very large values for elements of the estimated covariance matrix ${\Omega}_{\varvec{u}}^{c}$ for one of the classes. Hence, we selected the three-class growth mixture model with homogeneous error structure as the best fitting model.

**Fig. 5:**
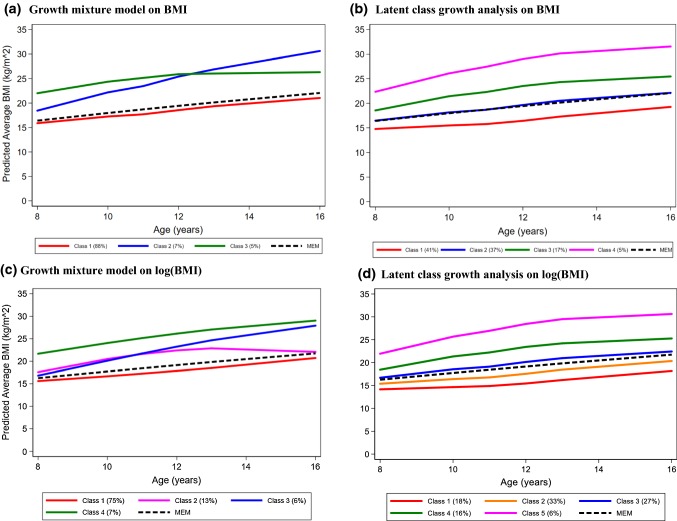
Best fitting trajectories of body mass index (BMI) obtained using a mixed effects model (MEM), a growth mixture model (left hand side panel) and a latent class growth analysis (right hand side panel) on the original BMI data (top) and log-transformed BMI (bottom); Avon Longitudinal Study of Parents and Children, N = 4517

The selected model’s predicted average trajectories are shown in Fig. 5a, together with the trajectory predicted by the mixed effects model (in black) for comparison. The class 1 trajectory (GMM-1, in red) is very similar to that predicted by the mixed effects model. This is not surprising given that class 1 is the most frequent, with 88% of the participants assigned to it according to their predicted posterior probability. Class 3 (GMM-3; 5%, in green) has a fairly parallel trajectory to that of GMM-1, albeit starting from a higher value. Class 2 (GMM-2; 7%, in blue) starts at a lower value than GMM-3 but increases faster over time, leading to the highest predicted BMI by age 16 years.

#### Latent class growth analysis

Six alternative specifications of latent class growth analysis model were fitted to the longitudinal BMI data. The four-class solution gave the best fit according to the goodness-of-fit criteria (green line in Fig. 4, Supplementary Table 4). The predicted trajectories for the four classes do not cross (Fig. 5b), unlike those identified by the best fitting growth mixture model. The second class (LCGA-2, in blue, 37%) overlaps with the trajectory predicted by mixed effects model (MEM; in black).

As expected, the lack of intra-class variability assumed by the latent class growth analysis model led to a greater number of classes than found by the best fitting growth mixture model. However, they also differed in shape. This might derive from biases affecting either model as a consequence of incorrect assumptions about the correlation structure of the BMI observations.

#### Longitudinal latent class analysis

The same modelling steps used to select the best growth mixture model and latent class growth analysis model were used when fitting longitudinal latent class analysis models. The best fitting model predicted identical trajectories to those obtained by latent class growth analysis. This is not surprising since the only difference between the two models is how time (here age) is accounted for: it is included as a continuous explanatory variable in the latent class growth analysis specification (and modelled here using a quadratic function) while it is an ordered categorical variable in longitudinal latent class analysis (see Supplementary Table 4).

#### Changing outcome scale

Each of the fitted models described above assumes that the residual errors are normally distributed, conditionally on class (in latent class growth and longitudinal latent class analysis) or on class *and* individual (for growth mixture models). If this assumption is inappropriate, results may be biased, with our conclusions regarding the number of latent classes potentially erroneous [40]. For this reason, we refitted all models on log-transformed BMI values, given its known skewness.

The new mixed effects model (i.e., the one-class GMM) showed a marked improvement in fit (in terms of AIC, BIC and c-BIC) relative to the mixed effects model fitted on the original scale (Supplementary Table 4). The best fitting growth mixture model fitted on the transformed data had four classes (with no gain in the fit indices when allowing class-specific covariance structures; Fig. 4). Interestingly, the most frequent classes of the growth mixture model fitted on the original and log-transformed values (GMM-1, in red, in Fig. 5a, c, respectively 88% and 75% of the total) have very similar trajectories (after back-transformation of the latter). However, the solutions differed with regards to the remaining classes, with the classes derived from the new model showing a separation of the individuals who start with moderate values: log-transforming the data on BMI before fitting the growth mixture model separated a group of individuals who continue to increase their BMI (GMM-3, in blue, 6%) from those whose increase slows down after age 12 (GMM-2, in pink, 13%).

The best latent class growth analysis model fitted on log-transformed BMI had five classes (Fig. 5d), with the new class showing a finer separation among the individuals. Again, the best fitting latent class growth analysis model has more classes than the best fitting growth mixture model, with the first three latent class growth analysis classes nearly completely overlapping with GMM-1 (hence capturing its distribution). However, the other classes do not follow this pattern (Supplementary Fig. 1).

**Fig. 6:**
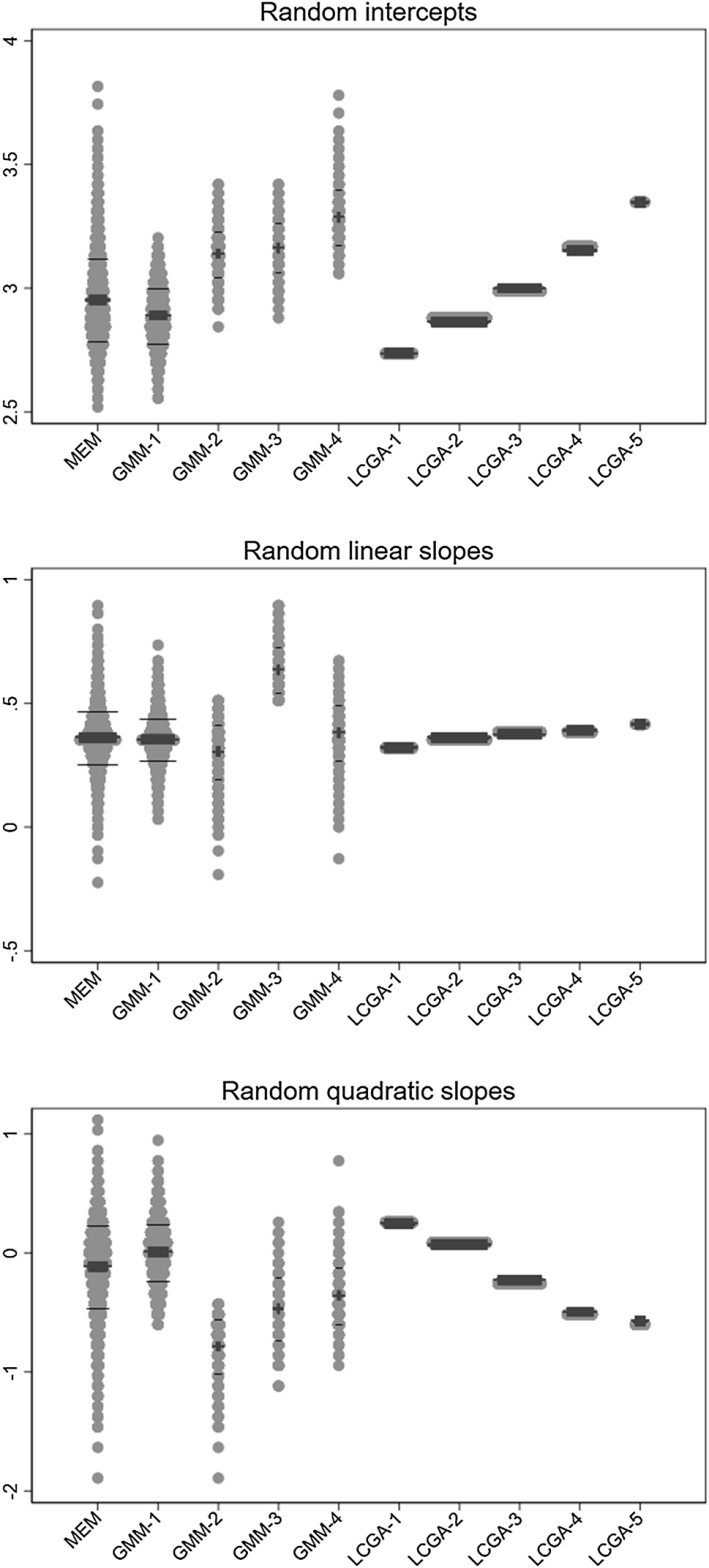
Distribution of the random coefficients predicted by alternative models, fitted to log-transformed body mass index (BMI); Avon Longitudinal Study of parents and children, N = 4517. *MEM* mixed effects model, *GMM* growth mixture model, *LCGA* latent class growth analysis; *GMM-n* nth class of GMM with 4 classes, *LCGA-n* nth class of LCGA model with 5 classes. Grey dots: observation, thick black line: median, thin black line: 1st and 3rd quartile

In order to further interrogate these results, Fig. 6 compares the distributions of the random intercepts, linear slopes, and quadratic slopes predicted by the mixed effects models fitted on log(BMI), with those of the equivalent random coefficients predicted by the growth mixture model with four classes and the latent class growth analysis model with five classes (although the latter—by definition—do not have any within-class heterogeneity). The skewed distributions of the random intercepts predicted by the mixed effects model are neatly separated into the four growth mixture model classes. In contrast, the class-specific intercepts given by the latent class growth analysis do not fully reflect the spread of the mixed effects model random intercepts, and do not capture at all its extreme values. Similar comments apply to the distributions of the predicted random slopes (especially the linear ones, Fig. 6). Examination of the estimated residuals from each of these models showed no particular skewness. There is therefore no direct evidence of departure from the models’ assumptions. Note however that all estimates were obtained assuming the missing mechanism was MAR. If this were not the case, estimates would be affected by selection bias in directions that cannot be predicted. We discuss this further in “Alternative estimation approaches”.

### Fussy eating

#### Mixed effects models

**Fig. 7:**
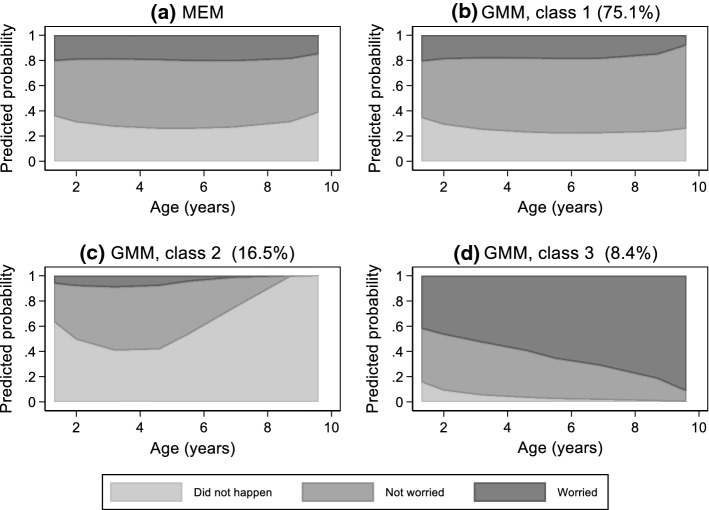
Stacked predicted probabilities of parental reports of their child’s fussy eating (“Did not happen”, “Not worried” and “A bit/greatly worried”) predicted by the best fitting mixed effects model (MEM) and the best fitting growth mixture model (GMM) with 3 classes; Avon Longitudinal Study of parents and children, N = 5824

Several mixed effects models for the longitudinal categorical fussy eating were fitted, with age capturing the dependence on time and with alternative specifications of their random components. The model with linear and quadratic terms for age and random intercepts, linear and quadratic slopes fitted the data best. The stacked predicted probabilities of parental reporting of, respectively, “Did not happen”, “Not worried”, and “A bit/greatly worried” are shown in Fig. 7a (details in Supplementary Table 6). They show stable probabilities over time of each category of parental reported fussy eating.

#### Growth mixture models

A three-class growth mixture model with homogeneous error structure (Ω_**u**_) was selected as the best fitting model, with attempts at relaxing this assumption resulting in no-convergence (Supplementary Table 7). According to this classification, most children (75.1%) were assigned to class 1 (GMM-1, Fig. 7b), which is characterised by predicted probabilities closely resembling those identified by the mixed effects model. The second most common class (GMM-2, 16.5%, Fig. 7c) comprises parents reporting that fussy eating “did not happen” with increasing predicted probabilities. The smallest class (GMM-3, 8.4%, Fig. 7d) includes children whose parents report worrying (a bit or greatly) with high and increasing probabilities, while “not worrying” and “did not happen” are reported with fast decreasing probabilities over time.

#### Latent class growth analysis

**Fig. 8:**
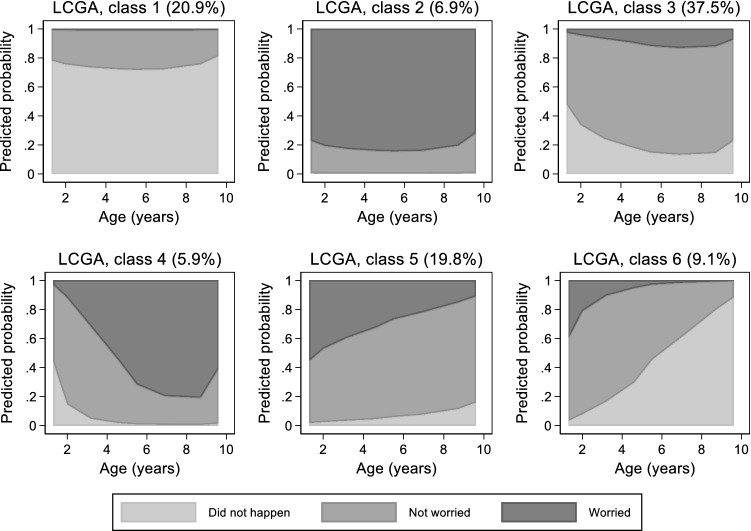
Stacked predicted probabilities of parental reports of their child’s fussy eating (“Did not happen”, “Not worried” and “A bit/greatly worried”) predicted by the best fitting latent class growth analysis (LCGA) with 6 classes; Avon Longitudinal Study of parents and children (ALSPAC) study, N = 5824

Latent class growth analysis of fussy eating suggested six classes fitted the data best (Supplementary Table 7). The cumulative predicted probabilities of each of the three categories varied substantially across classes (Fig. 8). Class 1 (LCGA-1; 20.9%) identifies a group of children whose parents report that fussy eating “did not happen” with high and fairly stable probabilities, while in class 2 (LCGA-2; 6.9%) parents worry (a bit or greatly) about their children’s fussy eating with fairly stable probabilities. In class 3 (LCGA-3; 37.5%) parents mostly do “not worry”. The other three classes show time-varying probabilities: LCGA-4 (5.9%) and LCGA-5 (19.8%) comprise parents that progressively increasingly and progressively decreasingly worry. LCGA-6 (9.1%) includes parents that progressively increasingly report that fussy eating “did not happen”.

#### Longitudinal latent class analysis

The best fitting longitudinal latent class analysis model also consisted of 6 classes (Supplementary Table 7). The shape of their predicted probabilities is very close to those derived from the best fitting latent class growth analysis. Again, the role of time seems to be well captured by the linear and quadratic terms used in the selected latent class growth analysis model.

#### Comparisons

**Fig. 9:**
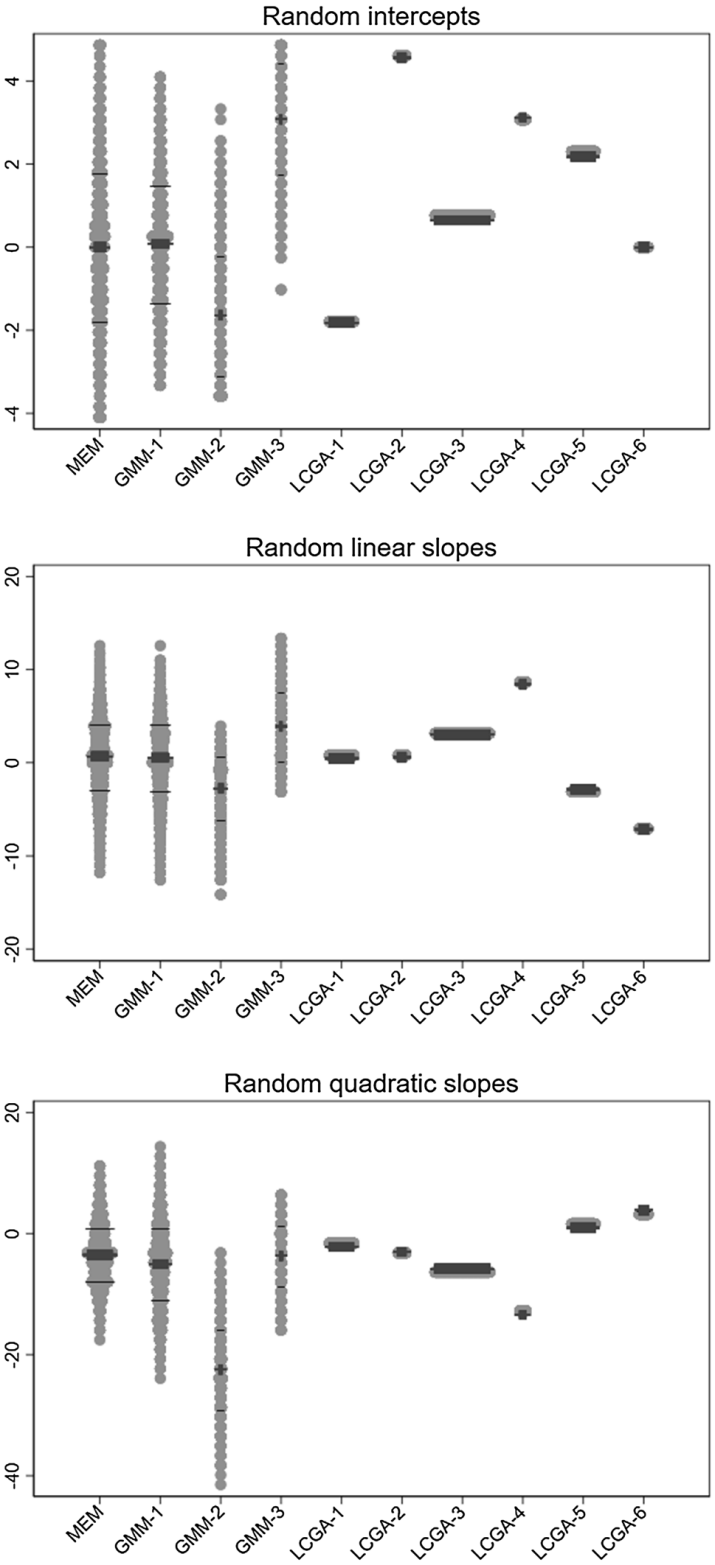
Distribution of the random coefficients predicted by alternative models fitted to fussy eating; Avon Longitudinal Study of parents and children, N = 5824. *MEM* mixed effects model, *GMM* growth mixture model, *LCGA* latent class growth analysis, *GMM-n* nth class of GMM with 4 classes, *LCGA-n* nth class of LCGA model with 6 classes. Grey dots: observation, thick black line: median, thin black line: 1st and 3rd quartile

The distributions of the random coefficients predicted by the mixed effects model, growth mixture model with three classes, and latent class growth analysis model with six classes are compared in Fig. 9. The distribution of random coefficients of the largest growth mixture model class (GMM-1) practically coincides with those for the mixed effects model, with the class-specific coefficients for LCGA-3 mirroring their means. In contrast, the distributions for GMM-2 and GMM-3 capture respectively the lower and upper tails of the mixed effects model distributions.There is a similar spread across the latent class growth analysis coefficients. The riverplot that links the classes predicted by the growth mixture with those from the latent class growth analysis model shows that LCGA-3 and LCGA-5 make up most of GMM-1 (all capturing large probabilities of being “not worried”), while LCGA-1 and LCGA-6 correspond to GMM-2 (large probabilities of “did not happen”), and LCGA-2 and LCGA-4 to GMM-3 (decreasing probabilities of “did not happen”; Supplementary Fig. 2). Hence, once it is recognised that the latent class growth analysis captures within-class variation by creating further classes, we find that the two modelling approaches lead to similar classifications.

### Associations with explanatory and outcome variables

The interpretation of the classes derived so far may be enhanced by relating them to precursors or later outcomes.

#### Association with birth weight

The best fitted models for log(BMI) and fussy eating were used to define their respective latent classes before relating them to birth weight (as in Fig. 2c). Multinomial logistic regression models were fitted where the probability of belonging to each class depended on this explanatory variable. Results are reported in terms of estimated relative risk ratios (RRR), i.e., ratios of the relative probability of being in a given class over the probability of being in the reference class, per 1 standard deviation (SD) increase in birth weight. We chose as reference the most frequent class from each growth mixture model; Class 1 for both longitudinal log(BMI) and fussy eating, and the closest latent class growth analysis class to these reference classes. These were respectively class 2 for log(BMI) and class 3 for fussy eating. We report results obtained using the 3-step approach first, before comparing them with those from the 1-step approach.

Birth weight was associated with an increased risk of being in the highest BMI growth mixture model class (GMM-4), relative to the reference (GMM-1), with an estimated 32% increase in relative risk [RRR = 1.32, 95% confidence interval (CI): 1.05, 1.68] per 1 SD increase in birth weight (Table 1, 3-step results). The estimated RRRs across the latent class growth analysis classes for BMI, relative to LGCA-2 (which trajectory is similar to GMM-1), show a negative association for the first class (LCGA-1) and a positive association with the other classes (Table 1). Of note is the similarity in RRRs for LCGA-5 and GMM-4. These results highlight, regardless of modelling approach, a positive association between birth weight and trajectories with persistently higher BMI.

**Table 1:** Estimated relative risk ratios (RRRs) and 95% confidence intervals (CI) of belonging to a given body mass index (BMI) or fussy eating (FE) class (relative to the reference class) per 1 SD increase in birth weight, estimated using either a 1-step or 3-step approach. The classes were identified using the best fitting growth mixture model (GMM) and best fitting latent class growth analysis (LCGA) model, for log(BMI) and FE; Avon Longitudinal Study of parents and children, N = 4227 for the BMI classes and N = 5437 for the FE classes

Variable	Model	Class^a^	1-step				3-step			
			Class %	RRR	95% CI		Class %	RRR	95% CI	
Log (BMI)	GMM	1 (ref)	74.8	1			74.7	1		
		2	12.4	1.17	0.96	1.42	12.6	1.06	0.86	1.31
		3	5.7	0.81	0.64	1.04	6.0	0.92	0.68	1.25
		4	7.2	1.45	1.21	1.76	6.7	1.32	1.05	1.68
	LCGA	1	18.2	0.74	0.67	0.81	17.9	0.77	0.70	0.84
		2 (ref)	33.0	1			33.3	1		
		3	27.1	1.10	1.00	1.21	27.3	1.13	1.02	1.24
		4	15.7	1.03	0.92	1.17	15.8	1.04	0.93	1.16
		5	5.9	1.25	1.05	1.48	5.8	1.30	1.09	1.55
FE	GMM^b^	1 (ref)	65.3	1			75.1	1		
		2	27.1	0.91	0.82	1.00	16.5	1.16	1.01	1.34
		3	7.6	1.12	0.86	1.46	8.4	0.96	0.83	1.10
	LCGA	1	20.7	0.99	0.89	1.10	20.9	1.04	0.93	1.16
		2	7.0	0.87	0.77	0.99	6.9	0.91	0.80	1.04
		3 (ref)	37.4	1			37.5	1		
		4	5.9	0.88	0.75	1.03	5.9	0.90	0.77	1.06
		5	19.9	0.88	0.80	0.97	19.8	0.90	0.81	1.00
		6	9.0	0.97	0.84	1.13	9.1	1.00	0.85	1.17

*BMI* body mass index, *FE* fussy eating; ref: reference

^a^As in Figs. 5, 7 and 8

^b^Results obtained after constraining the variance of the quadratic slope to be zero

With regards to the fussy eating classes, the RRRs for GMM-2 and LCGA-1 (relative to their respective, and comparable, reference classes) show increased relative risks with higher birth weight (Table 1, 3-step results). These two classes are characterised by large (GMM-2) and increasing (LCGA-1) frequencies of parental reporting that fussy eating “did not happen”. The opposite is seen with the classes characterised by increasing (GMM-3, LCGA-4) or large and stable (LCGA-2) parental worrying about fussy eating. Their RRR are all less than 1 (0.92-0.96). It appears therefore that fussy eating may be less commonly reported in children with greater birth weight.

#### Association with fat mass index

Similar steps were followed to examine the association between the BMI and fussy eating classes and FMI at age 18 years (Fig. 2d). Linear regression models were fitted on log-transformed FMI to address the right-skewness of its distribution.

The results for the BMI classes derived from the growth mixture and latent class growth analysis models are in agreement again, and in line with expectations, with larger average FMI associated with higher BMI trajectories (Table 2, 3-step results). Interestingly, GMM-3 and GMM-4 show similar differences in log-FMI, relative to GMM-1, both larger than those found for GMM-2 (characterized by relatively higher BMI but only initially), indicating the importance of BMI in later adolescence.

**Table 2:** Mean differences and 95% confidence intervals (CI) in fat mass index (FMI, log-transformed) across body mass index (BMI) and fussy eating (FE) classes (relative to the reference class) estimated using either a 1-step or 3-step approach. The classes were identified using the best fitting growth mixture model (GMM) and best fitting latent class growth analysis (LCGA) model respectively, for log(BMI) and FE, Avon Longitudinal Study of Parents and, N = 4227 for the BMI classes and N = 5437 for the FE classes

	Model	Class^a^	1-step				3-step			
			Class %	Dif.	95% CI		Class %	Dif.	95% CI	
Log (BMI)	GMM	1 (ref)	13.2	0			74.7	0		
		2	49.8	0.164	0.109	0.219	12.6	0.089	0.065	0.113
		3	28.6	0.330	0.273	0.387	6.0	0.297	0.275	0.319
		4	8.4	0.515	0.462	0.568	6.7	0.298	0.272	0.324
	LCGA	1	18.0	− 0.100	− 0.117	− 0.083	17.9	− 0.106	− 0120	− 0.092
		2 (ref)	33.2	0			33.3	0		
		3	27.2	0.095	0.077	0.113	27.3	0.099	0.087	0.112
		4	15.8	0.196	0.173	0.219	15.8	0.191	0.172	0.210
		5	5.9	0.314	0.282	0.346	5.8	0.307	0.280	0.334
FE	GMM	1 (ref)					75.1	0		
		2		^b^			16.5	0.023	− 0.002	0.048
		3					8.4	− 0.024	− 0.053	0.005
	LCGA	1	20.9	0.010	− 0.012	0.032	20.9	0.008	− 0.018	0.034
		2	6.9	− 0.027	− 0.054	0.000	6.9	− 0.031	− 0.064	0.002
		3 (ref)	37.4	0			37.5	0		
		4	5.9	− 0.210	− 0.055	0.013	5.9	− 0.024	− 0.069	0.021
		5	19.9	− 0.018	− 0.040	0.004	19.8	− 0.018	− 0.046	0.010
		6	9.0	0.009	− 0.023	0.041	9.1	− 0.003	− 0.041	0.035

*BMI* body mass index, *FE* fussy eating, *ref* reference, *Dif.* estimated mean difference

^a^As in Figs. 5, 7 and 8, except for 1-step GMM for log(BMI) which gave parallel trajectories (as opposed to those of Fig. 5)

^b^No results because of no convergence

The results for the fussy eating classes are less straightforward to interpret (Table 2, 3-step results). GMM-2, the growth mixture model class with decreasing reports of fussy eating, has greater average FMI than the reference class, GMM-1 (estimated difference = 0.047; 95% CI: -0.005, 0.099), while GMM-3, the class with increasing frequencies of parental worrying, has a smaller average FMI (-0.061; -0.132, 0.011).

Among the latent class growth analyses classes, only LCGA-2 has similar average FMI to the reference class LCGA-3, despite being characterised by very different trajectories, all the other LCGA classes having instead lower mean values. All these differences are however small and estimated with wide confidence intervals.

Overall, FMI at age 18 is on average higher in individuals that belong to classes with persistently high BMI values, as identified by the two modelling approaches as GMM-3 and LCGA-5. The growth mixture model gives an additional insight in identifying also GMM-2 as having the highest average FMI. This class has average BMI onset but the fastest increase over time. In contrast, no clear associations were found between FMI and the fussy eating classes derived from either modelling approach. This is likely a reflection of the complex consequences of fussy eating. Fussy children might only like a small variety of foods, some of which may have high caloric content [41].

#### Alternative estimation approaches

The results concerning the relationship between the explanatory/outcome variable and the latent classes were generally very similar when performing the 1-step or 3-step approach for latent class growth analysis. This was not the case when fitting growth mixture models. When relating birth weight with the growth mixture model classes, the 1-step approach identified different classes in comparison to the 3-step approach. This is a consequence of the impact of birth weight on the identification of the classes. When relating the growth mixture model classes to later FMI, we encountered no-convergence, even when the model was simplified by constraining the quadratic random effect to have zero variance. The derived classes were different between the 1-step and 3-step approach regardless.

To account for the possible departure from MAR, we also fitted both models conditionally on maternal education and maternal age at birth of the child (Supplementary Table 8). Similar to what happened with birth weight, this led to different frequencies of the latent classes when using the 1-step approach with growth mixture models. It therefore seems advisable to avoid using a 1-step approach when fitting growth mixture models.

## Final remarks

We have compared three different analytical approaches to derive latent trajectories from a variable observed longitudinally. In doing so we have reviewed the assumptions invoked when fitting these models (summarised in Table 3), and highlighted the importance of carefully evaluating them, because misspecifications may lead to biased estimates of the trajectories and to an overestimation of the number of classes. For this reason, any interpretation of the resulting classes needs to take into account possible sources of misspecification of the models and of the impact such misspecifications may have. Additionally, when describing the classes identified by latent class growth analysis (and its simplification, longitudinal latent class analysis), one should acknowledge their derivation as non-parametric representations of variation in the individual trajectories, as opposed to just (possibly substantive) underlying typologies.

**Table 3:** Overview of models that allow the investigation of latent trajectories from longitudinal data on a variable Z, where $Z_{ij}$ is observed on individual *i* at time $t_{j}$, with *i* = 1, 2, …, *N*, and *j *= 0, 1, …, *J*

Model	Assumptions	Comments
Growth mixture model	There are potentially multiple typical trajectories, called “classes” Within each class *c*, individual trajectories vary around the class mean trajectory according to how many random coefficients, ${\beta_{0i}^{c}}, {\beta_{1i}^{c}}, \ldots ,$ are specified and to how they are assumed to be correlated with each other (via $\Omega _{\varvec{u}}^{\varvec{c}}$) The class-specific trajectories are expressed as a function of time (using polynomials) Individual observations depart from the individual trajectory according to the distribution of $\varepsilon_{ij}^{c}$, which are assumed to be independent and normally distributed (or to follow a logistic distribution if $Z_{ij}$ is categorical) See Fig. 1b	Care should be taken in transforming $Z_{ij}$ to meet the model’s distributional assumptions Estimation is generally by ML + EM Given the complexity of the model’s specification, estimation may require considerable computing time, and may fail, especially when $Z_{ij}$ is categorical Examining the distribution of the predicted random effects may help the evaluation of the appropriateness of the model’s specification Examination of the distribution of the estimated residuals may help the assessment of the distributional and time function assumptions Examining the distribution of the predicted random effects against those from a mixed effects model may help the interpretation of the classes
Latent class growth analysis	There are potentially multiple typical trajectories, called “classes” Within each class *s*, individual trajectories are identical The class specific trajectories are expressed as a function of time (using polynomials) Individual observations depart from the class-specific trajectory according to the distribution of $e_{ij}^{s}$, which are assumed to be independent and normally distributed (or to follow a logistic distribution if $Z_{ij}$ is categorical) See Fig. 1c	Care should be taken in transforming $Z_{ij}$ to meet the model’s distributional assumptions Estimation is generally by ML + EM Estimation is generally very fast Examination of the distribution of the estimated residuals may help the assessment of the distributional and time function assumptions When too few classes are selected, the residual errors $e_{ij}^{s}$ are likely to be correlated, leading to biased estimates Examining the distribution of the class-specific parameters against those from mixed effects and growth mixture models may help the interpretation of the classes, separating those that capture within-typology from between-typology variation
Longitudinal latent class analysis	There are potentially multiple typical trajectories, called “classes” Within each class *r*, individual trajectories are identical The class specific trajectories are allowed to freely vary with time Individual observations depart from the class-specific trajectory according to the distribution of $e_{ij}^{r}$, which are assumed to be independent and normally distributed (or to follow a logistic distribution if $Z_{ij}$ is categorical) See Fig. 1d	Care should be taken in transforming $Z_{ij}$ to meet the model’s distributional assumptions Estimation is generally by ML + EM Estimation is generally faster than for the other models Examination of the distribution of the estimated residuals may help the assessment of the distributional assumptions When too few classes are selected, the residual errors $e_{ij}^{r}$ are likely to be correlated, leading to biased estimates Not parsimonious if the number of repeated observations *J* is large Examining the predicted trajectories against those from latent class growth analysis ones may help identify whether the relationship with time assumed in the latter should be modified

ML: maximum Likelihood; EM: expectation–maximization algorithm

Our view is that each of these modelling approaches offers a useful representation of the heterogeneities in individual trajectories and that much can be learnt from comparing results. We have found that starting the analyses by first fitting a mixed effects model to the data helps understanding the data and that much in gained by examining the correspondence across classes obtained from different models, and by locating the class-specific parameters estimated by latent class growth analysis within the distributions of predicted random effects from the corresponding mixed effects and latent growth models.

Comparing the strength and direction of the associations between the latent classes and both birth weight and fat mass index was enlightening for the understanding of the underlying typologies. Furthermore, assessing the support for an association between known predictors and the classes (or the classes and a subsequent outcome) offers insights into the typologies captured by the classes. However, much care should be invested in comparing results across models to avoid overinterpreting the results.

Generalizations of these models to more than one longitudinal variable are in principle straightforward, although they lead to complexities in both specification and estimation. Not surprisingly, even greater caution should accompany the interpretation of any resulting latent trajectories from multivariate longitudinal data.

In summary, this overview and the applications presented stress the importance of extensive and careful modelling, the advantages of comparing results across modelling approaches, and the need to temper the temptation of interpreting the classes derived by any of these models as confirmed phenotypes.

## Electronic supplementary material

Below is the link to the electronic supplementary material.

Supplementary material 1 (DOCX 94 kb)

## Abbreviations

ALSPAC: Avon longitudinal study of parents and children
BMI: Body mass index
GBTM: Group based trajectory models
GCM: Growth curve models
GMM: Growth mixture models
LCA: Latent class models
LCGM: Latent class growth models
LLCA: Longitudinal latent class models
MAR: Missing at random
MEM: Mixed effects models
ML: Maximum likelihood
SEM: Structural equation models
