# Supplementary material for: Identifying typical trajectories in longitudinal data: modelling strategies and interpretations
Source: Eur J Epidemiol. 2020 Mar 5;35(3):205–22. doi: 10.1007/s10654-020-00615-6 (PMC7154024; doi:10.1007/s10654-020-00615-6)
Supplement: Supplementary file 1 — Supplementary material 1 (DOCX 94 kb) [file 10654_2020_615_MOESM1_ESM.docx]

**Supplementary Table 1** Summary of longitudinal data on body mass index (BMI, kg/m^2^) and fussy eating (FE); Avon Longitudinal Study of Parents and Children

|  |  |  |  |  |
| --- | --- | --- | --- | --- |
| **BMI** | | | | |
|  | **N** | **Mean** | **SD** |  |
| **BMI at 8y** | 3,759 | 16.4 | 2.2 |  |
| **BMI at 10y** | 3,592 | 17.9 | 3.0 |  |
| **BMI at 11y** | 3,515 | 18.4 | 3.3 |  |
| **BMI at 12y** | 3,368 | 19.4 | 3.5 |  |
| **BMI at 13y** | 3,178 | 20.1 | 3.6 |  |
| **BMI at 16y** | 2,674 | 21.8 | 3.7 |  |
|  | | | | |
|  | **N** | **“No/did not happen”** | **“Not worried”** | **“A bit/greatly worried”** |
| **FE at 1.3y** | 5,215 | 2179 (42%) | 2057 (39%) | 979 (19%) |
| **FE at 2.0y** | 4,940 | 1513 (31%) | 2254 (46%) | 1173 (24%) |
| **FE at 3.2y** | 4,786 | 1252 (26%) | 2373 (50%) | 1161 (24%) |
| **FE at 4.6y** | 4,568 | 934 (20%) | 2729 (60%) | 905 (20%) |
| **FE at 5.5y** | 4,277 | 1081 (25%) | 2523 (59%) | 673 (16%) |
| **FE at 6.9y** | 4,014 | 1095 (27%) | 2247 (56%) | 672 (17%) |
| **FE at 8.7y** | 3,797 | 1266 (33%) | 1918 (51%) | 613 (16%) |
| **FE at 9.6y** | 3,759 | 1260 (34%) | 1948 (52%) | 551 (15%) |

y=years

**Supplementary Table 2** Number of available observations on longitudinal body mass index (BMI, kg/m^2^) and Fussy eating (FE); Avon Longitudinal Study of Parents and Children

| **No of BMI observations** |  | **BMI** | |  | |
| --- | --- | --- | --- | --- | --- |
|  | **N** | **Mean** | **SD** |  |  |
|  |  |  |  |  |  |
| 1 | 573 | 18.4 | 4.1 |  |  |
| 2 | 334 | 17.5 | 2.9 |  |  |
| 3 | 359 | 17.4 | 3.3 |  |  |
| 4 | 444 | 17.0 | 2.7 |  |  |
| 5 | 850 | 16.6 | 2.5 |  |  |
| 6 | 1,957 | 16.2 | 2.0 |  |  |
| At least one | 4,571 | 16.8 | 2.8 |  |  |
|  |  |  |  |  |  |
| **No of FE observations** |  | **FE** | | | |
|  | **N** | **“no/did not happen”** | **“not worried”** | **“a bit/greatly worried”** |  |
| 1 | 403 | 175 (43%) | 162 (40%) | 66 (16%) |  |
| 2 | 350 | 135 (39%) | 143 (41%) | 72 (21%) |  |
| 3 | 363 | 149 (41%) | 144 (40%) | 70 (19%) |  |
| 4 | 329 | 134 (41%) | 126 (38%) | 69 (21%) |  |
| 5 | 421 | 179 (43%) | 160 (38%) | 82 (19%) |  |
| 6 | 546 | 220 (40%) | 216 (40%) | 110 (20%) |  |
| 7 | 829 | 339 (41%) | 316 (38%) | 174 (21%) |  |
| 8 | 2,583 | 1048 (41%) | 1064 (41%) | 471 (18%) |  |
| At least one | 5,824 | 2379 (41%) | 2331 (40%) | 1114 (19%) |  |

**Supplementary Table 3** Fitted mixed effects models for longitudinal BMI and longitudinal log(BMI); Avon Longitudinal Study of Parents and Children, N=4,517

|  |  |  | **BMI** (kg/m^2^) | | | | | **Log BMI** (log kg/m^2^) | | | | | | |  |  |
| --- | --- | --- | --- | --- | --- | --- | --- | --- | --- | --- | --- | --- | --- | --- | --- | --- |
|  |  | Estimate | | SE | Random Effects  Covariance Matrix | | | Estimate | SE | | Random Effects  Covariance Matrix | | | | |  |
| Fixed effects | Intercept | 19.44 | | 0.05 | 12.40 |  |  | 2.95 | 0.003 | | 0.029 | | |  |  |  |
|  | Linear slope^(a)^ | 7.08 | | 0.06 | 10.35 | 6.04 |  | 0.36 | 0.003 | | 0.018 | | | 0.006 |  |  |
|  | Quadratic slope^(a)^ | -1.24 | | 0.17 | 70.57 | -18.96 | 1.44 | -0.11 | 0.008 | | 0.177 | | | 0.007 | -0.048 |  |
| Residual | Age 8 |  | |  | 0.23 |  |  |  |  | | 0.001 | | |  |  |  |
| Variances | Age 10 |  | |  | 0.47 |  |  |  |  | | 0.001 | | |  |  |  |
|  | Age 11 |  | |  | 0.41 |  |  |  |  | | 0.001 | | |  |  |  |
|  | Age 12 |  | |  | 0.59 |  |  |  |  | | 0.001 | | |  |  |  |
|  | Age 13 |  | |  | 1.33 |  |  |  |  | | 0.003 | | |  |  |  |
|  | Age 16 |  | |  | 0.16 |  |  |  |  | | 0.000 | | |  |  |  |
| AIC |  |  | |  | 72147.7 |  |  |  | |  | | | -47776.8 | | | |
| BIC |  |  | |  | 72243.9 |  |  |  |  | | | -47680.5 | | | |  |
| c-BIC |  |  | |  | 72196.2 |  |  |  |  | | | -47728.2 | | | |  |

AIC: Akaike Information Criterion; BIC: Bayesian Information Criterion; c-BIC: sample size corrected Bayesian Information Criterion

^(a)^Age was rescaled to be centred on 0 by subtracting mean age 12 years divided by 10.

**Supplementary Table 4**  Comparison of goodness of fit criteria for Growth Mixture Models and Latent Class Growth Analysis of longitudinal BMI data fitted on the original or log-transformed scale; Avon Longitudinal Study of Parents and Children, N=4,517, best-fitting solution in bold

| **Models** | **Scale** | **No. of classes** | **AIC** | **BIC** | **cBIC** | **Entropy** | **Sample size per class based on most likely class membership** |
| --- | --- | --- | --- | --- | --- | --- | --- |
| **GMM** | **Original scale** | 1 | 72147.659 | 72243.894 | 72196.229 | 1 |  |
|  |  | 2 | 71249.578 | 71371.475 | 71311.100 | 0.919 | 4192 (93%), 325 (7%) |
|  |  | **3** | **70810.384** | **70957.943** | **70884.858** | **0.889** | **3960 (88%), 314 (7%), 243 (5%)** |
|  |  | 4 | 70493.720 | 70666.941 | 70581.146 | 0.868 | 3726 (82%), 381 (8%), 327 (7%), 83 (2%) |
|  |  | 5 | 70272.622 | 70471.506 | 70373.000 | 0.844 | 2554 (79%), 368 (8%), 265 (6%), 259 96%), 71 (2%) |
|  | **Original scale,**  **class-specific Ω_u_** | 2 | 69796.567 | 69956.957 | 69877.517 | 0.626 | (32%), (68%) |
|  |  | 3 | 69386.058 | 69610.604 | 69499.388 | 0.575 | (41%), (15%), (44%) |
|  |  | 4 | 69296.562 | 69585.264 | 69442.272 | 0.601 | 1822 (40%), 1709 (38%), 150 (3%), 835 (18%) |
|  |  |  |  |  |  |  |  |
|  | **Log-transformed** | 1 | -47776.750 | -47680.516 | -47728.181 | 1 |  |
|  |  | 2 | -48261.959 | -48140.063 | -48200.438 | 0.847 | 4087 (90%), 430 (10%) |
|  |  | 3 | -48484.581 | -48337.022 | -48410.107 | 0.739 | 3472 (77%), 685 (15%), 360 (8%) |
|  |  | **4** | **-48635.171** | **-48461.950** | **-48547.745** | **0.747** | **3376 (75%), 567 (13%), 303 (7%), 270 (6%)** |
|  |  | 5 | -48725.139 | -48526.255 | -48624.761 | 0.756 | 3309 (73%), 561 (12%), 277 (6%), 243 (5%), 128 (3%) |
|  |  | 6 | -48755.777 | -48531.231 | -48642.447 | 0.756 | 3210 (71%), 467 910%), 414 (9%), 100 (2%), 41 (<1%) |
|  | **Log-transformed, class- specific Ω_u_** | 2 | -48833.962 | -48692.819 | -48762.727 | 0.580 | (32%), (68%) |
|  |  | **3** | **-48916.581** | -48730.528 | -48822.679 | 0.554 | 1244 (28%), 2389 (53%), 884 (20%) |
|  |  | 4 | -48981.966 | -48693.264 | -48836.257 | 0.561 | (1%), (47%), (20%), (12%) |
|  |  |  |  |  |  |  |  |
|  | **Non-Par** | 4 |  | -48263.256 |  | 0.86 | With 2 point masses- not a stable result |
|  |  |  |  |  |  |  |  |
| **LCGA** | **Original scale** | 1 | 102864.698 | 102922.438 | 102893.840 | 1 |  |
|  |  | 2 | 90863.373 | 90946.775 | 90905.466 | 0.896 | 3403 (75%), 1114 (25%) |
|  |  | 3 | 84380.359 | 84489.424 | 84435.404 | 0.883 | 2445 (54%), 1630 (36%), 442 (10%) |
|  |  | **4** | **80395.860** | **80530.588** | **80463.858** | **0.870** | **1863 (41%), 1652 (37%), 775 (17%), 226 (5%)** |
|  |  | 5 | 78077.871 | 78238.261 | 78158.821 | 0.853 | 1558 (34%), 1327 929%), 1005 (22%), 486 (11%), 140 (3%) |
|  |  | 6 | 76247.079 | 76433.132 | 76340.981 | 0.858 | 1474 (33%), 1107 (25%), 995 (22%), 618 (14%), 258 (6%), 55 (1%) |
|  | **Log-transformed** | 1 | -17471.373 | -17413.632 | -17442.231 | 1 |  |
|  |  | 2 | -29439.674 | -29356.271 | -29397.580 | 0.859 | 2962 (66%), 1555 (34%) |
|  |  | 3 | -35868.021 | -35758.955 | -35812.975 | 0.866 | 1981 (44%), 1862 (41%), 674 (15%) |
|  |  | 4 | -39549.097 | -39414.369 | -39481.099 | 0.861 | 1753 (39%), 1385 (31%), 1032 (23%), 346 (8%) |
|  |  | **5** | **-42171.025** | **-42010.635** | **-42090.075** | **0.857** | **1502 (33%), 1234 (27%), 806 (18%), 712 (16%), 262 (6%)** |
|  |  | 6 | -43628.002 | -43441.949 | -43534.100 | 0.853 | 1374 (30%), 1185 (26%), 762 (17%), 642 (14%), 415 (9%), 139 (3%) |
| **LLCA** | **Original scale** | 2 | 90828.322 | 90950.219 | 90889.844 | 0.896 | 3402 (75%), 1114 (25%) |
|  |  | 3 | 84311.940 | 84478.745 | 84396.127 | 0.884 | 2444 (54%), 1630 (36%), 442 (10%) |
|  |  | **4** | **80289.822** | **80501.537** | **80396.676** | **0.871** | **1865 (41%), 1652 (37%), 773 (17%), 227 (5%)** |
|  |  | 5 | 77952.822 | 78209.446 | 78082.342 | 0.854 | 1555 (37%), 1325 (29%), 1007 (22%), 489 (11%), 141 (3%) |
|  |  | 6 | 76077.134 | 76378.668 | 76229.320 | 0.859 | 1475 (33%), 1112 (25%), 990 (22%), 618 (14%), 267 (6%), 56 (1%) |
|  | **Log-transformed** | 1 | -17481.571 | -17404.584 | -17442.715 | 1 |  |
|  |  | 2 | -29489.520 | -29367.623 | -29427.998 | 0.859 | 2961 (66%), 1556 (34%) |
|  |  | 3 | -35958.522 | -35791.716 | -35874.334 | 0.867 | 1980 (44%), 1861 (41%), 676 (15%) |
|  |  | 4 | -39671.044 | -39459.329 | -39564.190 | 0.862 | 1749 (39%), 1389 931%), 1032 (23%), 347 (8%) |
|  |  | **5** | **-42344.631** | **-42088.007** | **-42215.111** | **0.858** | **1504 (33%), 1236 (27%), 805 (18%), 711 (16%), 262 (6%)** |
|  |  | 6 | -43823.894 | -43522.361 | -43671.708 | 0.854 | 1368 (30%), 1182 (26%), 766 (17%), 634 (14%), 427 (9%), 141 (3%) |

AIC: Akaike Information Criterion; BIC: Bayesian Information Criterion; c-BIC: sample size corrected Bayesian Information Criterion

^(a)^ Number of random starts increased to 1,000 with 40 optimization phases due to convergence issues.

^(b)^ Non-positive definite matrix because of negative slope variance.

^(c)^ Variance slope fixed to zero. d No convergence.

**Supplementary Figure 1** River plot of most likely class membership derived from the best growth mixture model (GMM) and latent class growth analysis (LCGA) models fitted on log-transformed BMI; Avon Longitudinal Study of Parents and Children, N=4,517.


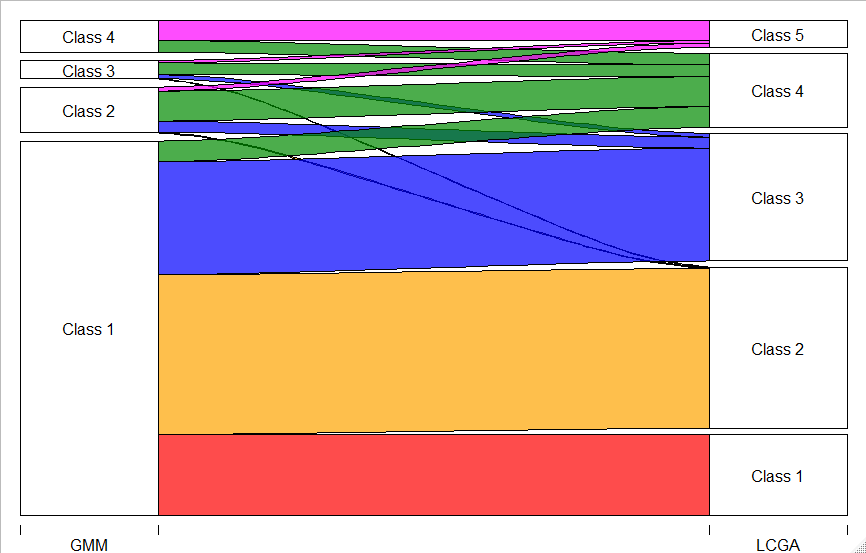


**Supplementary Table 6** Best fitted mixed effects model for longitudinal fussy eating; Avon Longitudinal Study of Parents and Children, N=5,824

|  |  |  | | | Random Effects | | |
| --- | --- | --- | --- | --- | --- | --- | --- |
|  |  | Estimate | SE | Variance- Covariance matrix^(b)^ | | | |
| Fixed effects | Intercept | 0.00 | - | 4.17 | |  |  |
|  | Linear slope^(a)^ | 0.67 | 0.10 | 0.13 | | 25.18 |  |
|  | Quadratic slope^(a)^ | -3.53 | 0.20 | -0.39 | | -31.35 | 50.56 |
| Thresholds | 1 | -1.73 | 0.04 |  | |  |  |
|  | 2 | 2.30 | 0.04 |  | |  |  |

^(a)^ Age was centred at 0 by subtracting mean age

^(b)^ Assumed to be homogenous across classes

**Supplementary Table 7** Comparison of goodness of fit criteria for growth mixture models (GMM) and latent class growth analysis (LCGA) models of longitudinal fussy eating; Avon Longitudinal Study of Parents and Children, N=5,824, best-fitting solution in bold

| **Models** | **No. of**  **classes** | **AIC** | **BIC** | **cBIC** | **Entropy** | **Sample size per class based on most likely class membership** |
| --- | --- | --- | --- | --- | --- | --- |
| **GMM** | 1 | 61640.689 | 61707.386 | 61675.609 | 1 |  |
|  | 2 | 61367.307 | 61460.683 | 61416.195 | 0.72 | 5164 (89%), 660 (11%) |
|  | **3** | **61321.085** | **61441.140** | **61383.941** | **0.67** | **4374 (75%), 960 (16%), 490 (7%)** |
|  | 4 |  |  |  |  | **No convergence** |
| **GMM** Class-specific Ω_u_ | 2 |  |  |  |  | **No convergence** |
|  | 3 |  |  |  |  | **No convergence** |
| **LCGA** | 1 | 71879.508 | 71906.187 | 71893.476 | 1 |  |
|  | 2 | 65615.821 | 65669.179 | 65643.757 | 0.678 | 2978 (50%), 2896 (50%) |
|  | 3 | 63522.206 | 63602.242 | 63564.110 | 0.700 | 3158 (54%), 1545 (27%), 1122 (19%) |
|  | 4 | 62550.649 | 62657.365 | 62606.522 | 0.663 | 1420 (42%), 1556 (27%), 975 (17%), 873 (15%) |
|  | 5 | 62148.731 | 62282.126 | 62218.571 | 0.668 | 2392 (41%), 1295 (22%), 938 (16%), 635 (11%), 563 (10%) |
|  | **6** | **61851.909** | **62011.983** | **61935.718** | **0.668** | **2182 (37%), 1215 (21%), 1512 (20%), 527 (9%), 403 (7%), 345 (6%)** |
|  | 7 | 61639.844 | 61826.596 | 61737.620 | 0.667 | 2070 (36%), 1187 (20%), 1074 (18%), 418 (7%), 377 (6%), 354 (6%), 344 (6%) |
|  | 8 | 61453.067 | 61666.498 | 61564.812 | 0.635 | 1893 (33%), 892 (15%), 797 (14%), 729 (13%), 411 (7%), 410 (7%), 349 (6%), 343 (6%) |
|  | 9 | 61346.164 | 61586.275 | 61471.877 | 0.639 | 1851 (32%), 830 (14%), 798 (14%), 758 (13%), 418 (7%), 337 (6%), 326 (6%), 301 (5%), 205 (4%) |
| **LLCA** | 1 | 71159.993 | 71266.709 | 71215.866 | 1 |  |
|  | 2 | 64467.387 | 64687.488 | 64582.624 | 0.680 | 2745 (47%), 3079 (53%) |
|  | 3 | 61912.492 | 62245.979 | 62087.094 | 0.686 | 2638 (45%), 1825 (33%), 1261 (22%) |
|  | 4 | 61355.445 | 61802.318 | 61589.411 | 0.661 | 2215 (38%), 1563 (27%), 1246 (21%), 801 (14%) |
|  | 5 | 60979.557 | 61539.815 | 61272.887 | 0.617 | 1677 (29%), 1150 (20%), 1088 (19%), 961 (16%), 947 (16%) |
|  | **6** | **60680.878** | **61354.522** | **61033.573** | **0.610** | **1550 (27%), 1032 (18%), 919 (16%), 843 (14%), 780 (13%), 700 (12%)** |
|  | 7 | 60471.609 | 61258.639 | 60883.669 | 0.608 | 1425 (24%), 970 (17%), 890 (15%), 758 (13%), 735 (13%), 713 (12%), 333 (6%) |
|  | 8 | 60352.692 | 61253.108 | 60824.117 | 0.601 | 1409 (24%), 826 (14%), 799 (14%), 767 (13%), 759 (13%), 496 (9%), 442 (8%), 326 (6%) |
|  | 9 | 60273.233 | 61287.034 | 60804.022 | 0.600 | 1419 (24%), 794 (14%), 696 (12%), 691 (12%), 533 (9%), 500 (9%), 449 (8%), 414 (7%), 328 (6%) |
|  | 10 | 61302.318 | 61569.108 | 61441.999 | 0.642 | 1895 (33%), 809 (14%), 744 (13%), 687 (12%), 355 (6%), 353 (6%), 325 (6%), 283 (5%), 201 (3%), 173 (3%) |

AIC: Akaike Information Criterion; BIC: Bayesian Information Criterion; c-BIC: sample size corrected Bayesian Information Criterion

**Supplementary Figure 2** River plot of most likely class membership derived from the best fitting growth mixture (GMM) and latent class growth analysis (LCGA) models fitted on fussy eating; Avon Longitudinal Study of Parents and Children, N=5,824.


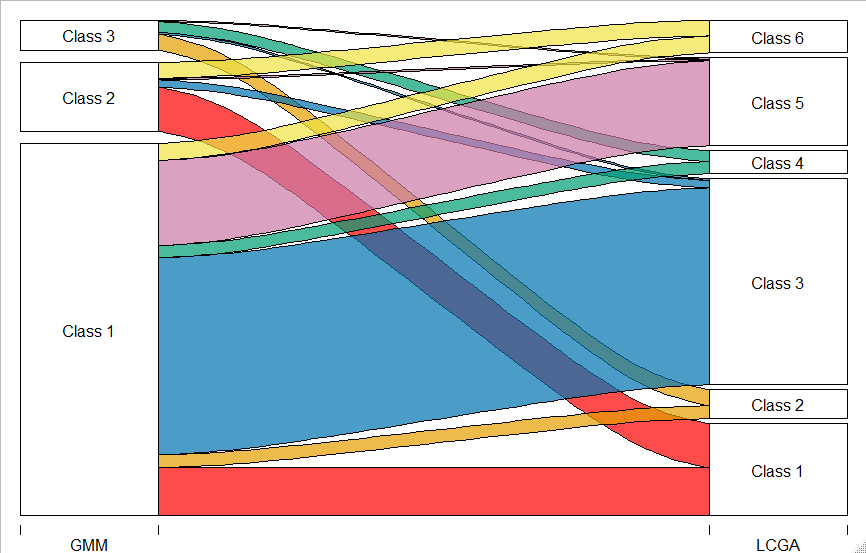


**Supplementary Table 8.**  Estimated relative risk ratios (RRRs) and 95% confidence intervals (CI) of belonging to a given body mass index (BMI) or fussy eating (FE) class (relative to the reference class) per 1 SD increase in birth weight, estimated using either a 1-step or 3-step approach, controlled for maternal education and maternal age. The classes were identified using the best fitting growth mixture model (GMM) and best fitting latent class growth analysis (LCGA) model, for log(BMI) and FE; Avon Longitudinal Study of Parents and Children, N=4,227 for the BMI classes and N=5,437 for the FE classes.

| **Variable** | **Model** | **Class†** | **1-step** | | | | **3-step** | | | |
| --- | --- | --- | --- | --- | --- | --- | --- | --- | --- | --- |
|  |  |  | **Class%** | **RRR** | **95% CI** | | **Class%** | **RRR** | **95% CI** | |
|  |  |  |  |  |  |  |  |  |  |  |
| **Log(BMI)** | **GMM** | 1 (ref) | 74.2 | 1 |  |  | 74.7 | 1 |  |  |
|  |  | 2 | 11.9 | 1.20 | 0.86, | 1.48 | 12.6 | 1.06 | 0.86, | 1.31 |
|  |  | 3 | 6.8 | 0.92 | 0.72, | 1.17 | 6.0 | 1.07 | 0.81, | 1.41 |
|  |  | 4 | 7.1 | 1.47 | 1.21, | 1.80 | 6.7 | 1.34 | 1.05, | 1.72 |
|  |  |  |  |  |  |  |  |  |  |  |
|  | **LCGA** | 1 | 18.1 | 0.76 | 0.69, | 0.84 | 17.9 | 0.78 | 0.71, | 0.86 |
|  |  | 2 (ref) | 33.0 | 1 |  |  | 33.3 | 1 |  |  |
|  |  | 3 | 27.0 | 1.13 | 1.02, | 1.25 | 27.3 | 1.14 | 1.03, | 1.27 |
|  |  | 4 | 15.9 | 1.10 | 0.97, | 1.24 | 15.8 | 1.09 | 0.97, | 1.22 |
|  |  | 5 | 6.0 | 1.31 | 1.10, | 1.55 | 5.8 | 1.37 | 1.14, | 1.65 |
|  |  |  |  |  |  |  |  |  |  |  |
